# Fibrome desmoplastique osseux: à propos d’un cas

**DOI:** 10.11604/pamj.2025.52.104.49437

**Published:** 2025-11-11

**Authors:** Latifa Doublali, Adnane Adnani, Meriem Belhouari, Mouna Bourhafour, Souha Sahraoui

**Affiliations:** 1 Centre Mohammed VI, Centre Hospitalier Universitaire Ibn Rochd, Casablanca, Maroc

**Keywords:** Fibrome desmoplastique osseux, radiographies standards, imagerie par résonance magnétique, anatomopathologie, cas clinique, Desmoplastic fibroma of bone, conventional radiographs, magnetic resonance imaging (MRI), histopathology, case report

## Abstract

Le fibrome desmoplastique ou fibrome desmoïde osseux est une tumeur osseuse bénigne rare. Il représente environ 0,1 à 0,3% des tumeurs osseuses. Il a été décrit pour la première fois par Jaffe en 1958. Il s'agit d'une tumeur à évolution lente, à malignité évolutive locale, ne donnant pas de métastase ni de transformation maligne. Pour le diagnostic radiologique, l'imagerie par résonance magnétique (IRM) est l'examen de choix pour évaluer l'extension locale au niveau de l'os et éventuellement des parties molles. Le diagnostic positif est histologique: la tumeur est constituée d'un tissu pauvre en fibroblastes et très riche en fibres de collagène et est identique à l'histologie des fibromes desmoïdés des parties molles. Le diagnostic histologique différentiel est parfois difficile, notamment avec les fibrosarcomes de bas grade. Le traitement de choix est chirurgical et consiste en une résection tumorale large à chaque fois que la localisation tumorale le permet. L'évolution après traitement est marquée par un taux de récidive locale élevé en cas d'exérèse intra-lésionnelle.

## Abstract (en)

Desmoplastic fibroma, also known as desmoid fibroma of bone, is a rare benign bone tumor, accounting for approximately 0.1 to 0.3% of all bone tumors. It was first described by Jaffe in 1958. This tumor has a slow progression with locally aggressive behavior, without metastasis or malignant transformation. For radiological diagnosis, magnetic resonance imaging (MRI) is the modality of choice to assess local extension within the bone and, when present, into the surrounding soft tissues. The definitive diagnosis is histological: the tumor is composed of tissue poor in fibroblasts and rich in collagen fibers, identical to the histology of desmoid fibromas of the soft tissues. Histological differential diagnosis can be challenging, particularly with low-grade fibrosarcomas. The treatment of choice is surgical, consisting of wide tumor resection whenever the location allows. A high rate of local recurrence characterises post-treatment evolution in cases of intralesional excision.

## Introduction

Le fibrome desmoplastique, ou fibrome desmoïde osseux, est une tumeur osseuse bénigne rare [1, 2]. Il a été décrit pour la première fois par Jaffe en 1958 [3]. Il existe une similitude histologique et macroscopique entre le fibrome desmoplastique et les fibromes desmoïdes des tissus mous. La définition de l'Organisation mondiale de la Santé est: « tumeur osseuse bénigne mais localement agressive qui est caractérisée par la formation abondante de fibres de collagène par les cellules tumorales »; le tissu tumoral est « pauvrement cellulaire et les noyaux sont de forme ovoïde ou allongée »; « la cellularité, le pléomorphisme et l'activité mitotique du fibrosarcome sont absents ». Un cas de fibrome desmoplastique osseux pris en charge dans notre service est rapporté ici pour identifier les problèmes diagnostiques et thérapeutiques spécifiques susceptibles [4].

## Patient et observation

**Information du patient:** un jeune homme âgé de 18 ans a consulté en mars 2016 pour une douleur de la cheville gauche à l'occasion d'une chute de son vélo.

**Figure 1:**
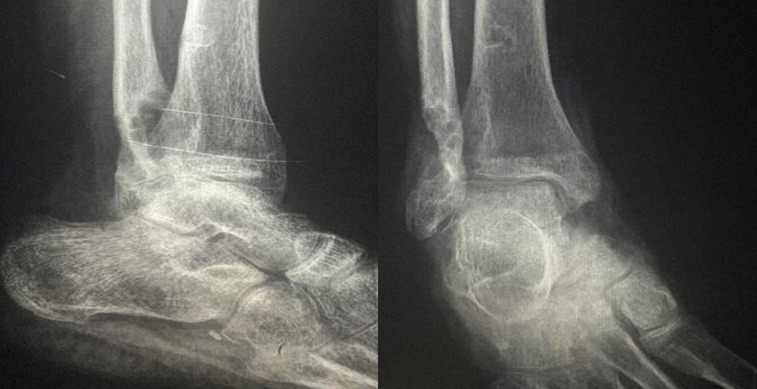
radiographie standard de la cheville gauche: lésion ostéolytique malléolaire externe, à contours flous, sans rupture corticale ni réaction périostée

**Figure 2:**
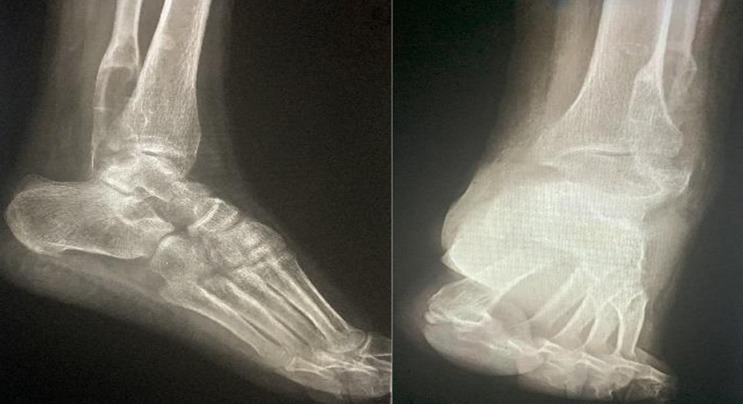
radiographie standard de la cheville gauche montrant une augmentation du volume de la lésion ostéolytique de la malléole externe, sans réaction périostée notable

**Figure 3:**
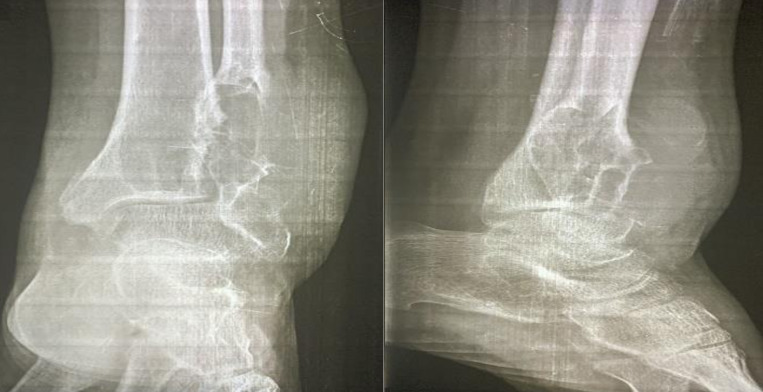
radiographie standard de la cheville gauche objectivant une lésion ostéolytique au niveau de la malléole externe avec rupture corticale partielle et contours irréguliers, envahissant légèrement les parties molles adjacentes

**Figure 4:**
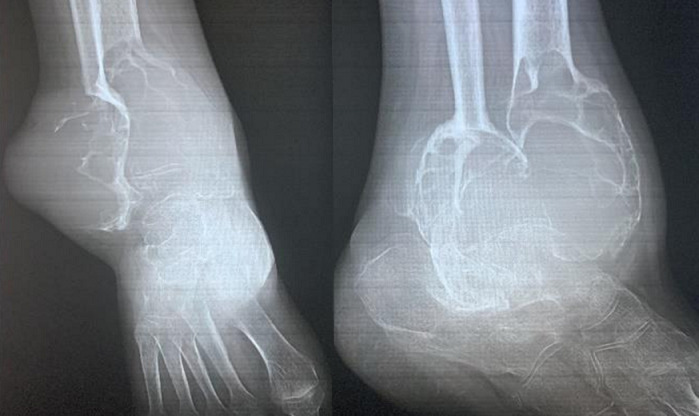
radiographie de la cheville gauche révélant une lésion ostéolytique extensive du quart distal du tibia et de la malléole externe, avec effraction corticale et extension dans les parties molles adjacentes

**Figure 5:**
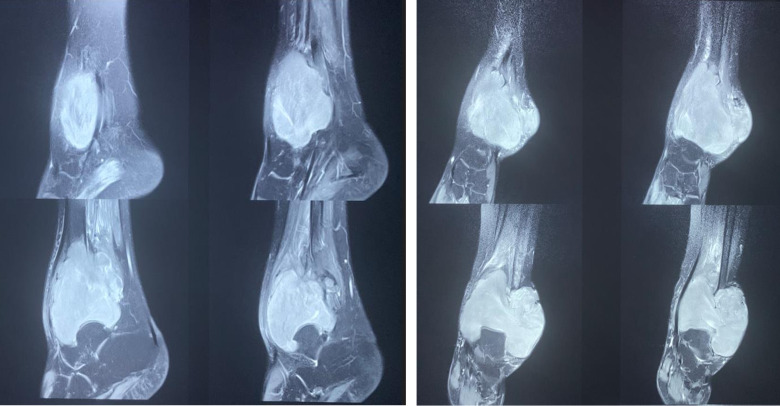
IRM de la cheville gauche montrant une masse métaphyso-épiphysaire bien limitée, iso-signal T1, hypersignal hétérogène T2, rehaussée de façon hétérogène après gadolinium, mesurant 114x79x98 mm

**Démarche diagnostique:** un bilan radiographique retrouvait une lésion ostéolytique de la malléole externe (Figure 1). Une biopsie osseuse était réalisée par un abord direct. L'examen histologique montrait une prolifération cellulaire dense à fond fibreux non atypique et peu atypique sans néoformation osseuse ni nécrose. Aucun diagnostic n'était retenu et une surveillance simple de cette lésion osseuse était donc décidée. En mars 2018, une radiographie montrait une augmentation du volume de la lésion (Figure 2). Une deuxième biopsie osseuse était réalisée et l'examen histologique montrait une prolifération tumorale à cellules myxoïdes et fusocellulaires faisant évoquer une fibromatose plantaire. La poursuite de la surveillance était décidée. En juillet 2021, une radiographie montrait une augmentation du volume de la lésion (Figure 3). Une troisième biopsie osseuse était réalisée. L'examen histologique montrait une prolifération fusocellulaire agencée en faisceaux courts enchevêtrés sur un fond collagénique parfois hyalinisé sans foyer de nécrose ni d'ostéogenèse. L'étude immunohistochimique montrait l'absence d'expression de PS100, P63, AML, CD34 et de béta-caténine. Le diagnostic histologique retenu était celui de fibrome desmoplastique osseux. La poursuite de la surveillance était donc décidée. En mai 2023, une radiographie montrait une augmentation du volume de la lésion (Figure 4). L'IRM de la cheville gauche retrouvait une masse tissulaire métaphyso-épiphysaire centrée sur l'extrémité distale du tibia et du péroné et l'articulation tibiopéronière distale gauche, bien limitée, de contours lobulés, en iso-signal T1, en hypersignal hétérogène T2, rehaussée de façon hétérogène après injection de gadolinium, mesurant 114x79x98 mm (Figure 5).

**Intervention thérapeutique et suivi:** l'intervention chirurgicale a été réalisée en juillet 2023. Le patient a bénéficié d'une amputation mi-jambe. L'examen histologique de la pièce d'exérèse concluait à «un fibrome desmoplastique osseux ». Le suivi postopératoire était simple.

**Consentement du patient:** le patient a donné son consentement éclairé pour la publication de ce rapport de cas, y compris les données cliniques, radiologiques, histologiques et les images associées.

## Discussion

Le fibrome desmoplastique apparaît comme une tumeur osseuse très rare. Elle représente, selon Taconis *et al*. [5], 0,3% des tumeurs osseuses bénignes. En 1958, Jaffe a distingué cette lésion osseuse des autres fibromes osseux, inventant le terme « fibrome desmoplastique » [3]. Selon Böhm *et al*. il n'existe pas de prédominance sexuelle et le fibrome desmoplastique survient dans plus de 75% des cas dans les trois premières décennies. Les sites les plus touchés sont la mandibule, le bassin et les os longs des membres inférieurs (fémur, tibia) [6]. Dans l'atteinte des os longs, la localisation tumorale est de préférence métaphysaire ; une extension épiphysaire, diaphysaire ou épiphysaire et diaphysaire est possible. Les signes cliniques sont variables et souvent discrets. Il s'agit d'une tumeur à évolution très lente et les symptômes révélateurs les plus fréquents sont la douleur et la tuméfaction, précédées pendant une longue période des troubles fonctionnels; une fracture pathologique est un symptôme annonciateur rare [6]. Notre observation témoigne également de cette lente évolution et de la discrétion des signes cliniques initiaux.

L'aspect radiographique du fibrome desmoplastique a été bien décrit par Taconis *et al*. [5]. Il s'agit d'une lésion ostéolytique, aux limites plus ou moins nettes, sans liséré dense péri-tumoral. Au sein de la tumeur, il existe un réseau de fines trabéculations osseuses. Ces trabéculations donnent un aspect décrit « en bulles de savon » ou « en nid d'abeille ». Une rupture corticale est observée dans 30% des cas mais ne doit pas être prise pour un signe de néoplasie. Cette solution de continuité corticale est mieux appréciée sur la tomodensitométrie [5]. Dans ce cas, on retrouve de façon quasi-constante une extension tumorale dans les parties molles adjacentes [6]. Une apposition périostée est rarement observée, en dehors des cas où il existe une fracture pathologique. Les constatations radiologiques de notre cas concordent bien avec ces descriptions. Devant l'aspect radiologique, plusieurs diagnostics différentiels peuvent être évoqués: tumeur à cellules géantes, kyste anévrysmal, kyste osseux solitaire, dysplasie fibreuse, fibrome non ossifiant, histiocytofibrome malin, fibrosarcome [5]. L'aspect IRM est caractérisé par la présence de zones en normo- et hyposignal en séquence T2. Les zones d'hyposignal correspondent à des zones hypocellulaires, très riches en tissu fibreux, et les zones présentant un signal plus intense correspondent à des zones plus cellulaires ou à des zones de nécrose [7]. L'IRM est l'examen de choix pour définir l'extension tumorale, à la fois intra-osseuse et extra-osseuse [6], pour la surveillance évolutive et le diagnostic d'une éventuelle récidive [5]. L'imagerie réalisée dans notre cas illustre bien l'intérêt de l'IRM pour préciser l'extension locale et le suivi évolutif. La sémiologie radiographique à l'IRM peut faire évoquer certains diagnostics différentiels : lymphome, fibrome non ossifiant, léiomyosarcome osseux primitif, dysplasie fibreuse et tumeur à cellules géantes [7]. La multiplicité des biopsies et la difficulté diagnostique rencontrée dans notre cas soulignent la complexité du diagnostic différentiel. La biopsie tumorale est un préalable thérapeutique indispensable. L'aspect macroscopique du fibrome desmoplastique a été bien décrit par Mazabraud [8]. Il s'agit d'un tissu homogène blanchâtre ou grisâtre de consistance ferme et élastique, ressemblant à du caoutchouc, la tranche de section de la tumeur apparaît brillante et fasciculée. Microscopiquement, il existe une grande similitude histologique du fibrome desmoïde osseux avec les fibromes desmoïdes des tissus mous (tumeurs desmoïdes abdominales ou extra-abdominales). L'histologie se caractérise par des faisceaux de fibres collagènes abondantes, formant souvent des stries hyalinisées, épaissies, séparées de manière égale par de rares fibroblastes fusiformes de petite taille, avec des noyaux petits, réguliers, ronds ou ovalaires, à chromatine fine, sans activité mitotique. Le fibrome desmoplastique présente une vascularisation modérée [8]. Il n'existe aucune composante osseuse ou cartilagineuse au sein de la tumeur. Avec l'immunohistochimie, les cellules sont réactives à un anticorps dirigé contre la vimentine [9]. L'aspect histologique observé dans notre cas, ainsi que les résultats immunohistochimiques, concordaient parfaitement avec les descriptions classiques rapportées dans la littérature. Le diagnostic différentiel histologique le plus délicat est le fibrosarcome de bas grade [5]. L'apparition de métastases fait redresser le diagnostic en faveur du fibrosarcome [10]. L'évolution observée confirme le caractère bénin et localement agressif du fibrome desmoplastique.

L'analyse du traitement du fibrome desmoplastique dans la littérature est difficile. En effet, la prise en charge thérapeutique repose sur l'expérience forcément limitée de chaque auteur, compte tenu de la rareté de cette tumeur. Néanmoins, il apparaît très clairement que les procédures intra-lésionnelles, type curetage, sont vouées à un taux de récidive très élevé, de 50 à 72% [3]. Les procédures de résection extra-lésionnelles offrent le meilleur résultat, avec moins de 5% de récidive [6]. La conduite thérapeutique adoptée dans notre patient s'inscrit dans cette logique de résection large afin de limiter le risque de récidive. Le fibrome desmoplastique reste cependant une tumeur bénigne et, suivant la localisation, une procédure de résection extra-lésionnelle de la tumeur peut engager le pronostic fonctionnel. La reconstruction après résection fait appel aux techniques habituelles de chirurgie tumorale: autogreffe vascularisée ou non, allogreffe, prothèse articulaire, etc. L'évolution naturelle du fibrome desmoplastique après traitement est marquée par le risque élevé de récidive tumorale. La récidive tumorale retrouve une tumeur présentant les mêmes caractéristiques histologiques que la tumeur initiale. En revanche, les cas décrits de transformation maligne en fibrosarcome sont le plus souvent considérés comme des sous-estimations de la nature exacte de la tumeur initiale. Il en est de même pour la survenue de métastases. L'absence de récidive à ce jour témoigne d'une évolution favorable après exérèse complète. Une surveillance postopératoire prolongée est recommandée, car les récidives surviennent souvent tardivement étant donné l'accroissement lent de la tumeur. En effet, le délai de survenue moyen des récidives est de trois ans. Des récidives ont été décrites jusqu'à dix ans après le traitement initial [6].

## Conclusion

Le fibrome desmoplastique est une tumeur osseuse bénigne, rare, à croissance lente et à malignité locale. Cette observation témoigne de la difficulté diagnostique et de la progression insidieuse de la lésion, nécessitant une corrélation clinique, radiologique et histologique. Le traitement repose sur une résection complète lorsque la localisation le permet, et la surveillance prolongée est essentielle pour prévenir et détecter toute récidive.
